# Crystal structure of (5-methyl­imidazo[1,2-*a*]pyridin-2-yl)methanol

**DOI:** 10.1107/S1600536814023022

**Published:** 2014-10-24

**Authors:** Abdelmalik Elaatiaoui, Mohammed Koudad, Rafik Saddik, Noureddine Benchat, Lahcen El Ammari

**Affiliations:** a Laboratory of Applied Chemistry and Environments (LCAE), Faculty of Sciences, University Mohammed Premier, Oujda, Morocco; b Laboratoire de Chimie du Solide Appliquée, Faculté des Sciences, Université Mohammed V-Agdal, Avenue Ibn Battouta, BP 1014, Rabat, Morocco

**Keywords:** crystal structure, imidazo[1,2-*a*]pyridine, hydrogen bonding, π–π inter­actions

## Abstract

In the title compound, C_9_H_10_N_2_O, the imidazo[1,2-*a*]pyridine moiety is approximately planar (r.m.s. deviation = 0.024 Å). The methanol group is nearly perpendicular to its mean plane as indicated by the C—C—C—O and N—C—C—O torsion angles of 80.04 (16) and −96.30 (17)°, respectively. In the crystal, mol­ecules are linked by O—H⋯N hydrogen bonds, forming inversion dimers with an *R* ^2^_2_(10) ring motif. The dimers are liked *via* C—H⋯O hydrogen bonds, enclosing *R* ^2^_2_(10) ring motifs and forming ribbons along [201]. The ribbons are linked *via* a number of π–π inter­actions [centroid–centroid distances vary from 3.4819 (8) to 3.7212 (8) Å], forming a three-dimensional structure.

## Related literature   

For the biological activities of derivatives of the title compound, see: Silvestre *et al.* (1998 ▶); Hamdouchi *et al.* (1999 ▶); Lhassani *et al.* (1999 ▶); Ertl *et al.* (2000 ▶). For the synthesis, see: Öhler *et al.* (1983 ▶); Chavignon *et al.* (1992 ▶).
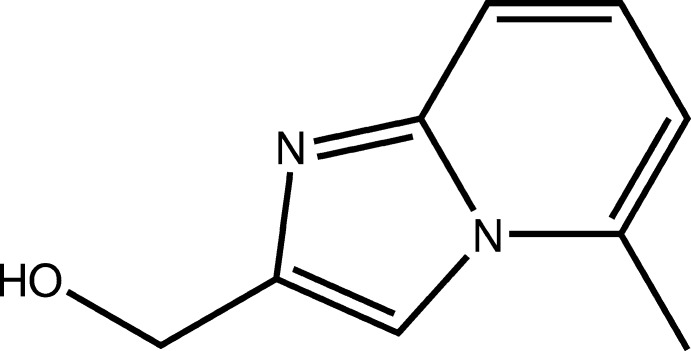


## Experimental   

### Crystal data  

C_9_H_10_N_2_O
*M* *_r_* = 162.19
Triclinic, 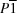

*a* = 7.3637 (2) Å
*b* = 8.1589 (2) Å
*c* = 8.3966 (2) Å
α = 62.355 (1)°
β = 67.291 (2)°
γ = 88.386 (2)°
*V* = 405.14 (2) Å^3^
*Z* = 2
Mo *K*α radiation
μ = 0.09 mm^−1^
*T* = 296 K
0.38 × 0.32 × 0.27 mm

### Data collection  

Bruker X8 APEX diffractometer
10226 measured reflections
2089 independent reflections
1865 reflections with *I* > 2σ(*I*)
*R*_int_ = 0.019

### Refinement  

*R*[*F* ^2^ > 2σ(*F* ^2^)] = 0.043
*wR*(*F* ^2^) = 0.123
*S* = 1.04
2089 reflections
110 parameters
H-atom parameters constrained
Δρ_max_ = 0.24 e Å^−3^
Δρ_min_ = −0.19 e Å^−3^

### 

Data collection: *APEX2* (Bruker, 2009 ▶); cell refinement: *SAINT* (Bruker, 2009 ▶); data reduction: *SAINT*; program(s) used to solve structure: *SHELXS97* (Sheldrick, 2008 ▶); program(s) used to refine structure: *SHELXL97* (Sheldrick, 2008 ▶); molecular graphics: *ORTEP-3 for Windows* (Farrugia, 2012 ▶); software used to prepare material for publication: *PLATON* (Spek, 2009 ▶) and *publCIF* (Westrip, 2010 ▶).

## Supplementary Material

Crystal structure: contains datablock(s) I. DOI: 10.1107/S1600536814023022/su5007sup1.cif

Structure factors: contains datablock(s) I. DOI: 10.1107/S1600536814023022/su5007Isup2.hkl

Supporting information file. DOI: 10.1107/S1600536814023022/su5007Isup3.cml

**Fig. 1.**
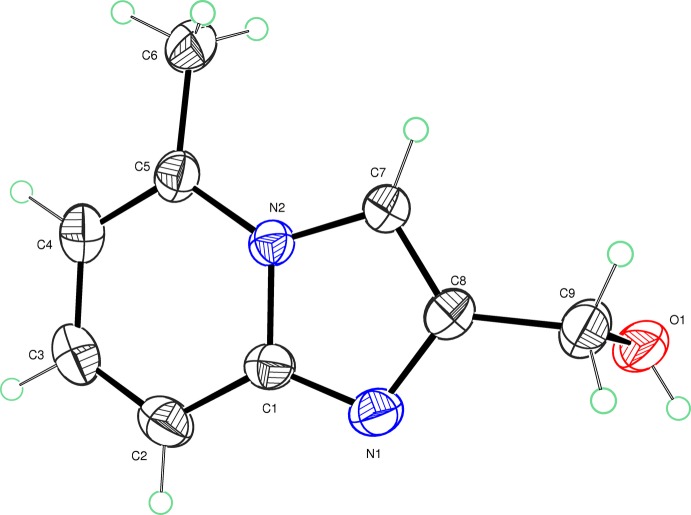
A view of the molecular structure of the title compound, with atom labelling. Displacement ellipsoids are drawn at the 50% probability level.

**Fig. 2.**
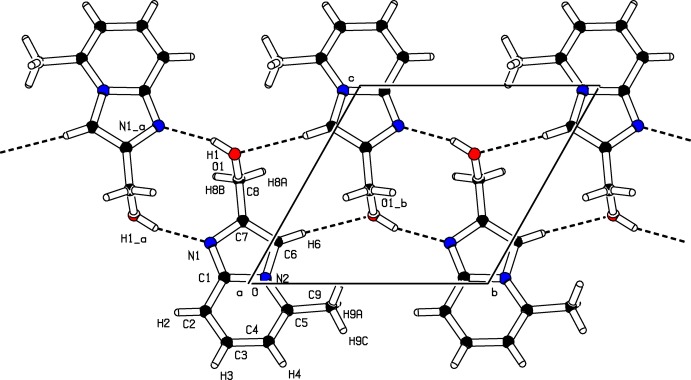
A partial view perpendicular to a axis of the crystal packing of the title compound, showing a layer of molecules linked by hydrogen bonds (dashed lines; see Table 1 for details).

. DOI: 10.1107/S1600536814023022/su5007fig1.tif
A view of the mol­ecular structure of the title compound, with atom labelling. Displacement ellipsoids are drawn at the 50% probability level.

a . DOI: 10.1107/S1600536814023022/su5007fig2.tif
A partial view perpendicular to *a* axis of the crystal packing of the title compound, showing a layer of mol­ecules linked by hydrogen bonds (dashed lines; see Table 1 for details).

CCDC reference: 1029873

Additional supporting information:  crystallographic information; 3D view; checkCIF report

## Figures and Tables

**Table 1:** Hydrogen-bond geometry (, )

*D*H*A*	*D*H	H*A*	*D* *A*	*D*H*A*
O1H1N1^i^	0.82	1.98	2.7734(17)	163
C6H6O1^ii^	0.93	2.55	3.4395(18)	160

Symmetry codes: (i) 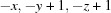; (ii) 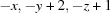.

## supplementary crystallographic information

### S1. Comment

Imidazo[1,2-*a*]pyridine moieties represent important building blocks in both natural and synthetic bioactive compounds, which have been shown to possess diverse therapeutic activities (Silvestre *et al.*, 1998; Hamdouchi *et al.*, 1999; Lhassani *et al.*, 1999; Ertl *et al.*, 2000). The synthesis of the title compound is based on the methods described in the literature (Ohler *et al.*, 1983; Chavignon *et al.*, 1992).

The title compound is formed by a fused five- and six-membered rings almost coplanar, with a maximum deviation of 0.029 (1) Å for C7 atom (Fig. 1). The mean plane through the fused ring system (N1/N2/C1-C7) is nearly perpendicular to the hydroxide group as indicated by the torsion angle C6–C7–C8–O1 of -96.30 (17) °.

The cohesion of the crystal structure is ensured by C6—H6···O1 and O1–H1···N1 hydrogen bonds, forming ribbons lying nearly perpendicular to the a axis, as shown in Fig. 2 and Table 1. There are a number of π-π interactions present linking the ribbons and forming a three-dimensional structure [Cg1···Cg1^i^ = 3.6025 (7) Å, Cg1··· Cg2^i^ = 3.6610 (8) Å, Cg1··· Cg2^ii^ = 3.7212 (8) Å, and Cg2··· Cg2^ii^ = 3.4819 (8) Å; where Cg1and Cg2 are the centroids of the N1/N2/C1/C6/C7 and N2/C1-C5 rings, respectively; symmetry codes: (i) -x , -y+2, -z; (ii) -x+1, -y+2, -z].

### S2. Experimental

The process for the synthesis of (5-methyl-imidazo[1,2-*a*]pyridine-2-yl) methanol described here occurs in two distinct stages: 1) Condensation of the 6-methylpyridin-2-amine with the ethyl bromopyruvate in boiling methanol. The mixture was then heated at 343 K for 4 h and neutralized at 273 K with Na_2_CO_3_. The product was extracted with dichloromethane. The organic layer was dried over sodium sulfate and the dichloromethane removed under reduced pressure. The crude product was purified on a silica gel column and identified as ethyl-5-methylimidazo [1,2-*a*]pyridine -2-carboxylate with 60% yield; 2) The reduction of the ester prepared above with lithium hydride and aluminium at room temperature in methanol for 2 h leads to a solid phase which was recrystallized from ethanol. Colourless crystals of the title compound were obtained with a yield of 67% (m.p. 413 K).

### S3. Refinement

H atoms were located in a difference Fourier map and treated as riding atoms with C–H = 0.93-0.98 Å, O–H = 0.82 Å and with *U*_iso_(H) = 1.5*U*_eq_ (C,O) for methyl and OH H atoms and = 1.2*U*_eq_(C) for other H atoms.

### Crystal data

**Table:**

C_9_H_10_N_2_O	*Z* = 2
*M**_r_* = 162.19	*F*(000) = 172
Triclinic, *P*1	*D*_x_ = 1.330 Mg m^−^^3^
Hall symbol: -p 1	Melting point: 413 K
*a* = 7.3637 (2) Å	Mo *K*α radiation, λ = 0.71073 Å
*b* = 8.1589 (2) Å	Cell parameters from 2089 reflections
*c* = 8.3966 (2) Å	θ = 2.9–28.7°
α = 62.355 (1)°	µ = 0.09 mm^−^^1^
β = 67.291 (2)°	*T* = 296 K
γ = 88.386 (2)°	Block, colourless
*V* = 405.14 (2) Å^3^	0.38 × 0.32 × 0.27 mm

### Data collection

**Table:**

Bruker X8 APEX diffractometer	1865 reflections with *I* > 2σ(*I*)
Radiation source: fine-focus sealed tube	*R*_int_ = 0.019
Graphite monochromator	θ_max_ = 28.7°, θ_min_ = 2.9°
φ and ω scans	*h* = −9→9
10226 measured reflections	*k* = −11→10
2089 independent reflections	*l* = −11→11

### Refinement

**Table:**

Refinement on *F*^2^	Secondary atom site location: difference Fourier map
Least-squares matrix: full	Hydrogen site location: inferred from neighbouring sites
*R*[*F*^2^ > 2σ(*F*^2^)] = 0.043	H-atom parameters constrained
*wR*(*F*^2^) = 0.123	*w* = 1/[σ^2^(*F*_o_^2^) + (0.0645*P*)^2^ + 0.0845*P*] where *P* = (*F*_o_^2^ + 2*F*_c_^2^)/3
*S* = 1.04	(Δ/σ)_max_ < 0.001
2089 reflections	Δρ_max_ = 0.24 e Å^−^^3^
110 parameters	Δρ_min_ = −0.19 e Å^−^^3^
0 restraints	Extinction correction: *SHELXL*, Fc^*^=kFc[1+0.001xFc^2^λ^3^/sin(2θ)]^-1/4^
Primary atom site location: structure-invariant direct methods	Extinction coefficient: 0.061 (14)

### Special details

**Table:**

Geometry. All e.s.d.'s (except the e.s.d. in the dihedral angle between two l.s. planes) are estimated using the full covariance matrix. The cell e.s.d.'s are taken into account individually in the estimation of e.s.d.'s in distances, angles and torsion angles; correlations between e.s.d.'s in cell parameters are only used when they are defined by crystal symmetry. An approximate (isotropic) treatment of cell e.s.d.'s is used for estimating e.s.d.'s involving l.s. planes.
Refinement. Refinement of *F*^2^ against all reflections. The weighted *R*-factor *wR* and goodness of fit *S* are based on *F*^2^, conventional *R*-factors *R* are based on *F*, with *F* set to zero for negative *F*^2^. The threshold expression of *F*^2^ > σ(*F*^2^) is used only for calculating *R*-factors(gt) *etc*. and is not relevant to the choice of reflections for refinement. *R*-factors based on *F*^2^ are statistically about twice as large as those based on *F*, and *R*- factors based on all data will be even larger.

### Fractional atomic coordinates and isotropic or equivalent isotropic displacement parameters (Å^2^)

**Table:**

	*x*	*y*	*z*	*U*_iso_*/*U*_eq_	
C1	0.23574 (17)	0.88694 (15)	0.02939 (17)	0.0374 (3)	
C2	0.3772 (2)	0.8861 (2)	−0.1413 (2)	0.0487 (3)	
H2	0.3890	0.7743	−0.1456	0.058*	
C3	0.49565 (19)	1.0507 (2)	−0.29887 (19)	0.0502 (3)	
H3	0.5907	1.0510	−0.4110	0.060*	
C4	0.47630 (19)	1.22086 (19)	−0.29465 (18)	0.0466 (3)	
H4	0.5601	1.3318	−0.4038	0.056*	
C5	0.33794 (18)	1.22657 (16)	−0.13469 (16)	0.0399 (3)	
C9	0.3049 (2)	1.39916 (18)	−0.1173 (2)	0.0565 (4)	
H9B	0.3236	1.3850	−0.0049	0.085*	
H9A	0.1709	1.4183	−0.1004	0.085*	
H9C	0.3987	1.5057	−0.2355	0.085*	
C6	0.07560 (16)	1.02021 (15)	0.21002 (15)	0.0363 (3)	
H6	0.0311	1.1066	0.2529	0.044*	
C7	0.01244 (16)	0.82991 (15)	0.31517 (16)	0.0383 (3)	
C8	−0.1332 (2)	0.71513 (18)	0.52658 (18)	0.0496 (3)	
H8A	−0.2211	0.7936	0.5652	0.060*	
H8B	−0.2151	0.6140	0.5406	0.060*	
N1	0.10972 (15)	0.74701 (14)	0.20387 (15)	0.0420 (3)	
N2	0.21944 (13)	1.05816 (12)	0.02646 (13)	0.0339 (2)	
O1	−0.03554 (17)	0.63769 (13)	0.65406 (14)	0.0566 (3)	
H1	−0.0705	0.5229	0.7180	0.085*	

### Atomic displacement parameters (Å^2^)

**Table:**

	*U*^11^	*U*^22^	*U*^33^	*U*^12^	*U*^13^	*U*^23^
C1	0.0427 (6)	0.0355 (5)	0.0412 (6)	0.0108 (4)	−0.0231 (5)	−0.0201 (4)
C2	0.0558 (7)	0.0532 (7)	0.0519 (7)	0.0214 (6)	−0.0269 (6)	−0.0342 (6)
C3	0.0463 (6)	0.0690 (9)	0.0402 (6)	0.0161 (6)	−0.0178 (5)	−0.0310 (6)
C4	0.0450 (6)	0.0538 (7)	0.0341 (5)	0.0009 (5)	−0.0155 (5)	−0.0170 (5)
C5	0.0445 (6)	0.0379 (6)	0.0335 (5)	0.0009 (4)	−0.0176 (5)	−0.0134 (4)
C9	0.0760 (9)	0.0358 (6)	0.0433 (6)	−0.0035 (6)	−0.0164 (6)	−0.0147 (5)
C6	0.0392 (5)	0.0342 (5)	0.0336 (5)	0.0073 (4)	−0.0146 (4)	−0.0158 (4)
C7	0.0388 (5)	0.0343 (5)	0.0381 (5)	0.0057 (4)	−0.0178 (4)	−0.0139 (4)
C8	0.0488 (7)	0.0407 (6)	0.0411 (6)	0.0028 (5)	−0.0115 (5)	−0.0119 (5)
N1	0.0494 (6)	0.0332 (5)	0.0445 (5)	0.0086 (4)	−0.0222 (4)	−0.0179 (4)
N2	0.0376 (5)	0.0325 (4)	0.0329 (4)	0.0063 (3)	−0.0171 (4)	−0.0151 (4)
O1	0.0880 (7)	0.0361 (5)	0.0419 (5)	0.0043 (4)	−0.0303 (5)	−0.0136 (4)

### Geometric parameters (Å, º)

**Table:**

C1—N1	1.3299 (15)	C9—H9A	0.9600
C1—N2	1.3884 (14)	C9—H9C	0.9600
C1—C2	1.4126 (17)	C6—C7	1.3631 (15)
C2—C3	1.356 (2)	C6—N2	1.3815 (13)
C2—H2	0.9300	C6—H6	0.9300
C3—C4	1.407 (2)	C7—N1	1.3735 (15)
C3—H3	0.9300	C7—C8	1.4907 (16)
C4—C5	1.3576 (17)	C8—O1	1.4154 (16)
C4—H4	0.9300	C8—H8A	0.9700
C5—N2	1.3823 (14)	C8—H8B	0.9700
C5—C9	1.4857 (18)	O1—H1	0.8200
C9—H9B	0.9600		
			
N1—C1—N2	110.57 (10)	H9B—C9—H9C	109.5
N1—C1—C2	131.10 (11)	H9A—C9—H9C	109.5
N2—C1—C2	118.32 (11)	C7—C6—N2	105.70 (10)
C3—C2—C1	119.09 (12)	C7—C6—H6	127.1
C3—C2—H2	120.5	N2—C6—H6	127.1
C1—C2—H2	120.5	C6—C7—N1	111.22 (10)
C2—C3—C4	120.76 (12)	C6—C7—C8	127.34 (11)
C2—C3—H3	119.6	N1—C7—C8	121.36 (11)
C4—C3—H3	119.6	O1—C8—C7	111.84 (10)
C5—C4—C3	121.41 (12)	O1—C8—H8A	109.2
C5—C4—H4	119.3	C7—C8—H8A	109.2
C3—C4—H4	119.3	O1—C8—H8B	109.2
C4—C5—N2	117.59 (11)	C7—C8—H8B	109.2
C4—C5—C9	125.31 (11)	H8A—C8—H8B	107.9
N2—C5—C9	117.10 (10)	C1—N1—C7	105.62 (9)
C5—C9—H9B	109.5	C6—N2—C5	130.27 (10)
C5—C9—H9A	109.5	C6—N2—C1	106.89 (9)
H9B—C9—H9A	109.5	C5—N2—C1	122.79 (10)
C5—C9—H9C	109.5	C8—O1—H1	109.5

### Hydrogen-bond geometry (Å, º)

**Table:**

*D*—H···*A*	*D*—H	H···*A*	*D*···*A*	*D*—H···*A*
O1—H1···N1^i^	0.82	1.98	2.7734 (17)	163
C6—H6···O1^ii^	0.93	2.55	3.4395 (18)	160

Symmetry codes: (i) −*x*, −*y*+1, −*z*+1; (ii) −*x*, −*y*+2, −*z*+1.
