# Supplementary figures and images for: Crystal structure of (5-methyl­imidazo[1,2-a]pyridin-2-yl)methanol
Source: Acta Crystallogr Sect E Struct Rep Online. 2014 Oct 24;70(Pt 11):o1189–90. doi: 10.1107/S1600536814023022 (PMC4257347; doi:10.1107/S1600536814023022)

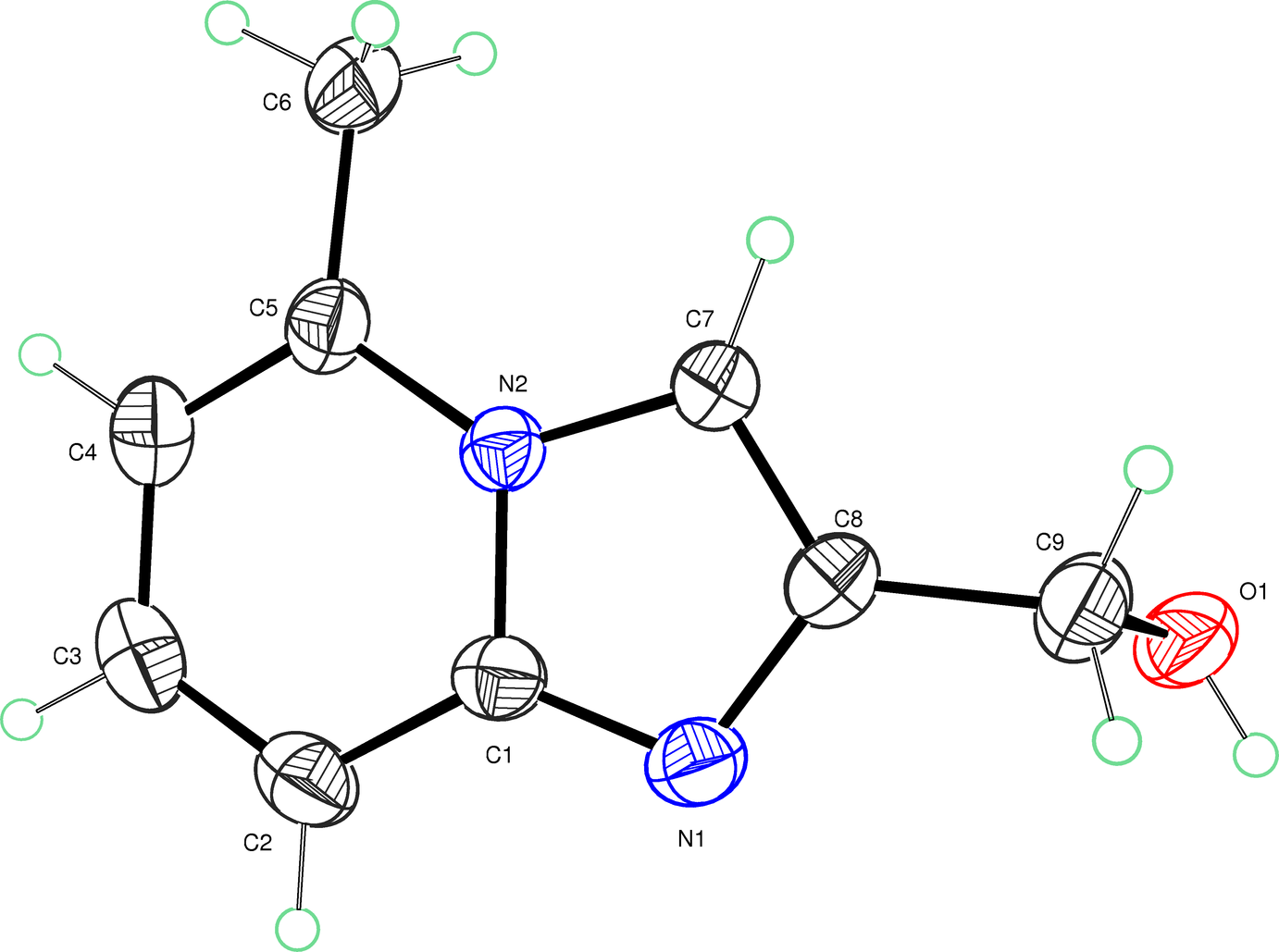

Supplement: Supplementary file 4 [file e-70-o1189-fig1.tif]

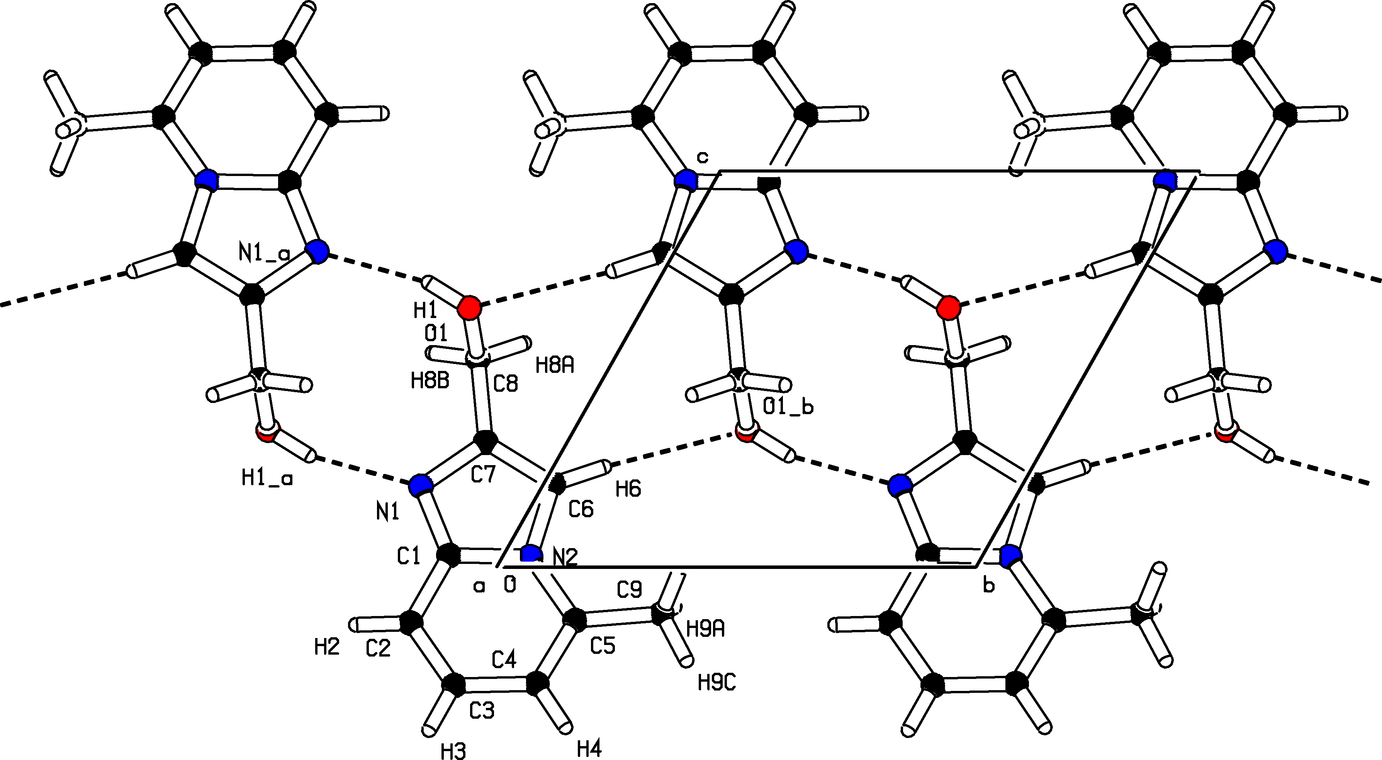

Supplement: Supplementary file 5 [file e-70-o1189-fig2.tif]
